# Diagnostic Accuracy of Fluorescein Sodium for Targeted Cervical Biopsies

**DOI:** 10.31557/APJCP.2021.22.7.2135

**Published:** 2021-07

**Authors:** Priya Bhati, Anitha Thomas, Ajit Sebastian, Thenmozhi Mani, Rachel G Chandy, Sudabeh Moein, Abraham Peedicayil

**Affiliations:** 1 *Gynaecologic Oncology, Christian Medical College, Vellore, Tamil Nadu, India. *; 2 *Biostatistics, Christian Medical College, Vellore, Tamil Nadu, India.*; 3 *Moein Health Foundation, CA, USA. *

**Keywords:** Cervix, screening, colposcopy, biopsy, fluorescence

## Abstract

**Background:**

Visual inspection methods for cervical cancer screening are widely used in low resource settings. Fluorescent sodium could improve accuracy of cancer screening. This study aimed to assess diagnostic accuracy of fluorescein sodium (FNa) to detect cervical neoplasia.

**Methods:**

Seventy consecutive patients referred for colposcopy were enrolled prospectively. Acetic acid, Lugol’s iodine, and FNa were used sequentially. Biopsies were taken from all abnormal areas. If there was no obvious abnormality, two random biopsies and endocervical curettage were done. Reference standard was the highest grade lesion on cervical biopsy with a threshold of CIN2+. The patterns of each staining agent were recorded as absent, faint, or distinct. Diagnostic accuracy estimates with 95% confidence intervals were calculated. Correlation between the various tests were also determined using the kappa statistic.

**Results:**

There were 27 cases of CIN2+ (38.6%). The sensitivity of any fluorescence for CIN2+ was 82% (62, 94) and for distinct fluorescence was 59% (39, 78). The specificity was 65% (49, 79) for any fluorescence and 95% (84, 99) for distinct fluorescence, the same as for Swede score > 7. For any fluorescence, the positive likelihood ratio was 2.34 (1.5, 3.65) and the negative likelihood ratio was 0.28 (0.13, 0.65). For distinct fluorescence, the positive likelihood ratio was 12.74 (3.18, 51.1) and the negative likelihood ratio was 0.43 (0.27, 0.68). There was moderate correlation between FNa and the other tests.

**Conclusion:**

Distinct fluorescence with FNa was very specific, low cost, and easy to perform and may contribute to confirm CIN2+ disease.

## Introduction

Cervical cancer is the fourth most common gynaecological malignancy worldwide in terms of both incidence and mortality (Bray et al., 2018). The cervix is amenable to screening by several methods which include visual inspection, cervical cytology, and human papillomavirus (HPV) DNA test. Colposcopically directed biopsies of the cervix can confirm the presence of cervical neoplasia. 

Low resource settings still rely on tests that are low cost, need little expertise, and are easy to perform by grass root health workers such as visual inspection with acetic acid (VIA) and visual inspection with Lugols iodine (VILI). However, these tests are subjective, require periodic training, are not useful in postmenopausal women, and have variable sensitivity and specificity (Sankarnarayanan et al., 2004). Cytology is labor intensive and requires trained laboratory personnel. Very sensitive tests, such as HPV DNA, lead to large numbers of women referred for colposcopy or over treatment in a screen and treat program . Thus, an accurate triage test is needed to overcome these problems.

Fluorescein sodium (C_20_H_12_O_5_Na_2_) is a dye made principally from two petroleum products called resorcinol and phthalic anhydride. Peak excitation occurs at 494 nm and peak emission at 521 nm, resulting in fluorescence.

Previous research has demonstrated that fluorescein sodium (FNa) accumulates preferentially in malignant tissue. Since 1881, FNa has been used as a fluorescent dye in a wide range of applications in clinical medicine, including ophthalmology (Haining, 1996; Yeh and Wilson, 2010, Xiao et al., 2010), neurosurgery (Kirchner and Proud, 1996; Su et al., 2013; Chen et al., 2012), urology (Cipolla et al., 1953; Van den Berg et al., 2012), general surgery (Haynes et al 1960), and colposcopy (Moein and Mohajer, 2016). FNa is used in these fields as a measure of tissue viability and a tool for the detection of neoplasia. 

The aim of this study was to determine the diagnostic accuracy of FNa in comparison to acetic acid, Lugol’s iodine, and colposcopy.

## Materials and Methods

The research proposal was approved by the institutional review board (ECR/326/INST/TN/2013) before commencement of the study. Written informed consent was obtained from all the participants.

Role of funding source: The study was supported by institutional research funding and the institution did not have any vested interest in the study. 

Sample size calculation: The required sample size to find an anticipated sensitivity and specificity of 90% with precision of 10% and 95% confidence, would be 35.

**Figure 1:**
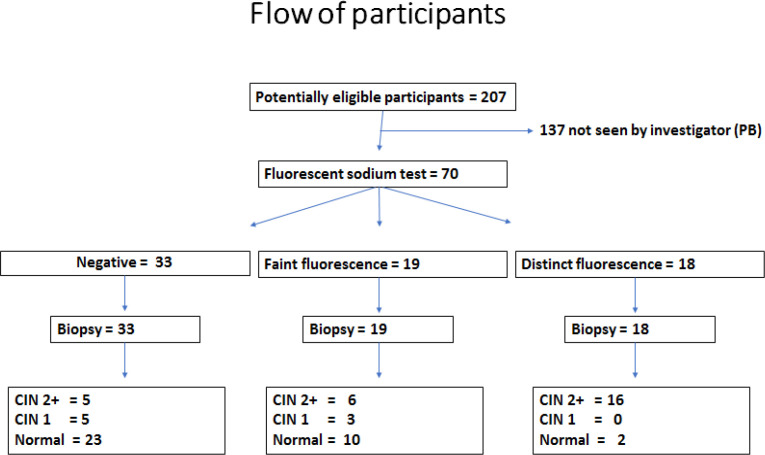
STARD Diagram of Participant Flow

Sample selection: Patients with abnormal screening test / abnormal symptoms who presented to our clinic between April 2018 to March 2019 were invited to participate in this study. Seventy consecutive patients seen by the first author (PB) and met inclusion criteria were recruited. There were no refusals (Figure 1).

Inclusion criteria: Women who fulfilled all of the following criteria were recruited into the study: i) age between 25-65 years, ii) having abnormal screen (Pap smear ASCUS or worse / HPV / VIA) results or referral for colposcopy for inter-menstrual or postcoital bleeding, and iii) being willing to participate in the study. Fifty-three patients (76%) out of 70 had abnormal cytology, of whom 27 were positive for high risk HPV DNA based on hybrid capture (Table 1).

Exclusion criteria: Women who had any of the following criteria were excluded: age under 25 or above 65 years, having menstrual bleeding, vaginal infection, sodium fluorescein allergy, or hypersensitivity, and being pregnant. 

Study design: This assessed the accuracy of a diagnostic test using a cross-sectional design. 

Study procedure: All colposcopic examinations and application of stains were done by the same investigator (PB). After discussing the study procedures and obtaining informed consent, the patient was placed in lithotomy position. A bivalve speculum was used to expose the cervix, and cervical mucus was removed with normal saline. Acetic acid solution (5%) was applied to the cervix for a minute. The cervix was visualized using a Borze 3500 colposcope (Borze Healthcare, New Delhi) for the transformation zone, squamocolumnar junction, acetowhite lesions, vascular patterns, and lesion size. All findings were documented on a standard diagram of the cervix. The cervix was again washed with normal saline and then Lugol’s iodine was applied. Mahogany brown or mustard yellow staining was noted and documented. The Swede score (Bowring et al, 2010) was assigned for each case. The cervix was again cleaned with saline.

Index or experimental test: FNa solution was prepared by dissolving 3 ophthalmic FNa strips (1 mg each) in 5 cc of sterile normal saline, resulting in a transparent yellow solution (off label use). This FNa solution was applied directly to the cervix and the cervix was washed with normal saline after 60 seconds. The room was darkened, and the cervix was then observed under UV light. If there was mucus, it would take up the stain so had to be removed . An orange filter made the fluorescence stand out. Fluorescence was recorded as ‘absent’, ‘faint’, seen best with an orange filter, and ‘distinct’ if strikingly obvious. The findings were documented on a standard diagram of the cervix. Fluorescent green colouring indicated abnormal areas. The test results were categorized as absent / faint / and distinct fluorescence. 

Comparison with other tests: The results of the standard colposcopic stains, fresh 5% acetic acid, and Lugol’s iodine were categorized as absent, faint, or distinct staining. The Swede score was used (minimum 0, maximum 10) with standard cut-offs of < 4, 5-6 and ≥ 7. For comparison, it was decided a priori that any (faint or distinct) fluorescence / staining as well as just distinct fluorescence / staining would be evaluated. 

Reference test: Abnormal areas of the cervix were marked on a clockface diagram of the cervix. Cervical biopsies of the areas stained by any of the three stains (FNa, Lugol’s iodine or acetic acid) were performed using a Tischler forceps on the same day as the index test. If there was no overlap in the various stains, biopsies were taken from each abnormal area. If there was no staining, random biopsies were taken from the anterior and posterior lips of the cervix (from transformation zone). Endocervical curettage was done if the transformation zone extended into the endocervical canal. The reference (gold) standard was the highest degree of abnormality of the cervical biopsy specimens. Although CIN1+ and CIN3+ were also considered, it was decided a priori that high grade cervical intraepithelial neoplasia or worse (CIN2+) would be the disease of interest. CIN2+ is the generally accepted reference category in the literature on screening for cervical neoplasia as it warrants immediate treatment.

Blinding: The person performing the index tests was blind to the biopsy result which usually was available only after 7 to 10 days. The pathologists were aware of the clinical information but were blinded to the results of the index tests.

 Statistical methods: The analysis was carried out using SPSS version 21.0 (Armonk, NY; IBM Corporation). For the main analysis, disease threshold for biopsy was “CIN2+ Two levels of test cut-offs (any positive and distinct positive) were taken for acetic acid, iodine, and FNa. For the Swede score, cut-offs taken were > 5 and > 7. 

Descriptive measures, such as mean with standard deviation (SD), were calculated for all continuous variables; whereas, frequencies and percentages were calculated for all categorical variables. Chi-square was used for test of association with alpha error of 5% and two-sided significance. Confidence intervals (95%) were estimated for the various diagnostic test characteristics. There was no missing information on index or reference tests.

Sensitivity, specificity, positive predictive value (PPV), negative predictive value (NPV), and likelihood ratios (LR) were calculated for fluorescein sodium, acetic acid, and Lugol’s iodine as staining agents taking histopathological findings as the gold standard. Agreement beyond chance between the various tests was assessed by Kappa values.

## Results

Patient characteristics: The mean age of the patients was 45.37 ± 11.18 years. The median parity was 2 (range 0-7). The mean BMI was 26.81 ± 4.01 kg/m^2^. All patients reported that they had only one sex partner. Only 2 patients were HIV seropositive (Table 1).

Indications for colposcopy: The reason for colposcopic examination was cytological abnormality in most of the patients. Thirty-one out of 70 (44.3%) patients had a Pap smear with high-grade squamous intraepithelial lesion (HSIL), while 19 (27.1%) had atypical cells or a low-grade lesion. High risk HPV DNA testing was available for 47 patients, and 24 were positive (51.1%).

Colposcopy results: Most patients had an adequate colposcopy with type I transformation zone (Table 2). Colposcopy was normal in 20 patients, while the others had a lesion with one or more of the stains. The Swede score was ≤ in 35 (50%), 5 or 6 in 17 (24.3%), and 7 to 10 in 18 patients (25.7%). 

Biopsy results: The reference test, biopsy, was normal in 31 patients (Table 3). Four patients had evidence of human papillomavirus infection. Cervical intraepithelial neoplasia (CIN1) was seen in 8 patients. CIN2+ was seen in 27 patients, of whom 8 had microinvasive or early invasive cervical cancer. Endocervical biopsy was normal in 16, CIN1 in 11, and CIN2+ in 14 patients.

FNa results: There were 18 patients with distinct fluorescence, 19 with faint fluorescence, and 33 with no fluorescence. No adverse effects were reported. Any fluorescence (faint or distinct) was significantly associated with biopsy result of CIN1+ (Table 4). Distinct fluorescence was also significantly associated with CIN2+ on biopsy. The sensitivity of any fluorescence to detect CIN2+ was 81.5% and specificity was 65.1%. FNa missed 3 cases of CIN2+ that was detected by endocervical curettage.

The sensitivity of distinct fluorescence to detect CIN2+ lesions was 59.3% and specificity was 95.4%. For any fluorescence, the LR was 2.34 (95% CI 1.5, 3.65), and the negative LR was 0.28 (95% CI 0.13, 0.65). For distinct fluorescence, the positive LR was 12.74 (95% CI 3.18, 51.1) and the negative LR was 0.43 (95% CI 0.27, 0.68). Distinct fluorescence showed moderate correlation (kappa 0.46 to 0.5) with acetic acid, iodine, and Swede score >5. 

**Figure 2:**
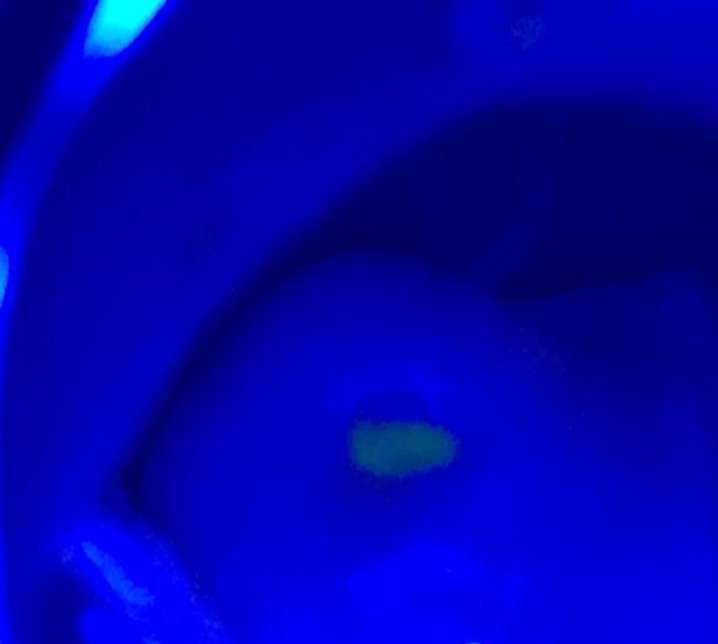
Absent Fluorescence after FNa Application under Ultraviolet Light

**Figure 3:**
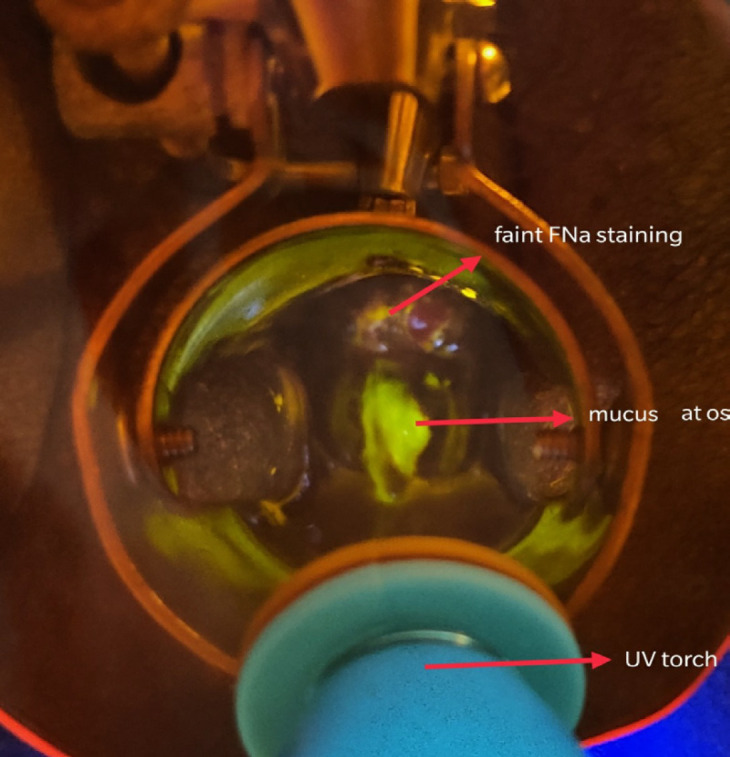
Fluorescence with Orange Filter after FNa Application under Ultraviolet Light

**Figure 4:**
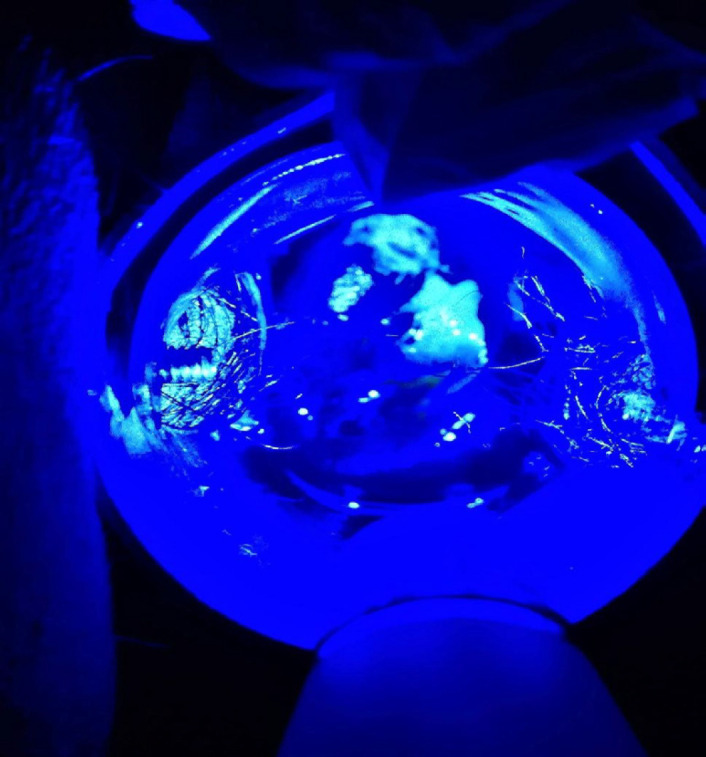
Distinct Fluorescence after FNa Application under Ultraviolet Light (No Filter)

The sensitivity of any fluorescence to detect CIN3+ was 76.2% and specificity was 57.1%. The sensitivity of distinct fluorescence to detect CIN3+ lesions was 52.4% and specificity was 85.7%. The absence of fluorescence is shown in Figure 2, faint fluorescence in Figure 3, and distinct fluorescence in Figure 4.

Comparison of index tests: Table 5 shows the diagnostic test characteristics against the reference standard of CIN2+ on biopsy. Any acetowhite, any iodine yellow, and Swede score < 4 were the most sensitive tests with sensitivity > 95% and a negative LR of < 0.1. Distinct fluorescence and a Swede score >7 were the most specific tests with specificity > 95% and positive LR (+LR) of 12.7. The best test results to rule out CIN2+ disease were no iodine staining, no acetowhite, and Swede score < 3. The best test results to rule in CIN2+ were distinct fluorescence and Swede score > 7. 

The percentage agreement for distinct fluorescence and Swede score > 5 was 70%. The kappa coefficient for Swede score > 5 and distinct fluorescence was 0.4 (p<0.001). 

The percentage agreement for distinct fluorescence and dense acetowhite was 80%. The kappa coefficient for dense acetowhite and distinct fluorescence was 0.495 (p<0.001). 

The percentage agreement for distinct fluorescence and mustard yellow on Lugol’s iodine was 80%. The kappa coefficient was 0.502 (p<0.001). 

**Table 1:** Baseline Characteristics of Women Referred for Colposcopy (N=70)

Characteristic	Frequency	Percentage
Age (years)		
< 30	3	4.3
30 – 50	43	61.4
> 50	24	34.3
Parity		
< 2	15	21.4
2 – 3	37	52.9
> 3	15	21.4
Missing	3	4.3
Haemoglobin (g/dl)		
< 8	2	2.8
8 – 11	23	32.9
> 11	36	51.4
Not tested	9	12.9
Human Immunodefficiency Virus (HIV)		
Positive	2	2.8
Negative	31	44.3
Not tested	37	52.9
Human Papilloma Virus (HPV)		
Positive	27	38.6
Negative	24	34.3
Not tested	19	27.1
Reason for colposcopy		
Clinical	17	24.3
Koilocytosis with high risk HPV	1	1.4
Abnormal Pap smear	52	74.3
Cytology		
Koilocytosis	1	1.4
ASCUS	10	14.3
LSIL	9	12.9
ASC-H or HSIL	31	44.3
AGC	2	2.8
Negative	17	24.3

**Table 2:** Findings on Women Referred for Colposcopy (N=70)

Colposcopic finding	Frequency	Percentage
Adequate	62	88.6
Inadequate	8	11.4
Transformation zone		
Type I	61	87.1
Type II	5	7.1
Type III	4	5.7
Ectopy	3	4.3
Nabothian cysts	3	4.3
Gland openings		
Normal	15	21.4
Cuffed	2	2.9
Quadrants involved		
None	20	28.6
One	12	17.1
Two	16	22.9
Three	14	20
Four	8	11.4
Swede Score		
≤ 4	35	50
5-6	17	24.3
≥ 7	18	25.7
Ectocervical biopsy	70	100
Endocervical curettage	41	58.6

**Table 3:** Biopsy Results on Patients Referred for Colposcopy (N=70)

Biopsy result	Frequency (n)	Percentage
Normal	31	44.3
Koilocytosis / HPV lesion	4	5.7
CIN I	8	11.4
CIN II	6	8.6
CIN III	13	18.6
Microinvasive cancer	3	4.3
Invasive cancer	5	7.1

**Table 4:** Cross Tabulation of Fluorescent Sodium with Two Biopsy Thresholds

Threshold 1	BIOPSY CIN 1+	BIOPSY < CIN 1	Total
Any	25	12	37
Fluorescence^a^			
No	10	23	33
fluorescence			
Total	35	35	70
Distinct	25	12	37
Fluorescence^b^			
No / faint	10	23	33
fluorescence			
Total	35	35	70
Threshold 2	BIOPSY CIN 2+	BIOPSY < CIN 2	Total
Any	22	15	37
Fluorescence^b^			
No	5	28	33
fluorescence			
Total	27	43	70
Distinct	16	2	18
Fluorescence^b^			
No / faint	11	41	52
fluorescence			
Total	27	43	70
Threshold 3	BIOPSY CIN3+	BIOPSY < CIN 3	Total
Any	16	21	37
Fluorescence^c^			
No	5	28	33
fluorescence			
Total	21	49	70
Distinct	11	7	18
Fluorescence^d^			
No / faint	10	42	52
fluorescence			
Total	21	49	70

^a^P=0.002; ^b^p<0.001; ^c^p=0.01; ^d^p=0.001

**Table 5:** Diagnostic Test Characteristics of Staining Agents for Diagnosis of CIN 2+

LEVEL of TEST / STAIN	Sens 95% CI	Spec 95% CI	AUC 95% CI	LR+ 95% CI	LR- 95% CI	PPV 95% CI	NPV 95% CI
Any acetowhite	96.3 (81 - 100)	46.5 (31 - 62)	0.71 (0.6 - 0.8)	1.8 (1.4 - 2.4)	0.1 (0.0 - 0.6)	53.1 (38 - 68)	95.2 (76 - 100)
Dense acetowhite	55.6 (35 -75)	88.4 (75 - 96)	0.72 (0.6 - 0.8)	4.8 (2.0 - 11.6)	0.6 (0.3 - 0.8)	75.0 (51 - 91)	76.0 (62 - 87)
Any iodine yellow	100.0 (87 - 100)	34.9 (21 - 51)	0.67 (0.6 - 0.8)	1.5 (1.2 - 1.9)	0.0 (0.0)	49.1 (35 - 63)	100.0 (78 - 100)
Distinct iodine yellow	70.4 (50 - 86)	86.1 (72 - 95)	0.78 (0.7 - 0.9)	5.0 (2..3 - 11)	0.3 (0.2 - 0.6)	76.0 (55 - 91)	82.2 (68 - 92)
Swede score > 5	81.5 (62 - 94)	69.8 (54 - 83)	0.76 (0.6 - 0.9)	2.7 (1.7 - 4.4)	0.3 (0.1 - 0.6)	62.9 (45 - 79)	85.7 (70 - 95)
Swede score > 7	59.3 (39 - 78)	95.4 (84 - 99)	0.77 (0.7 - 0.9)	12.7 (3.2 - 51.1)	0.4 (0.3 - 0.7)	88.9 (65 - 99)	78.9 (65 - 89)
Any fluorescence	81.5 (62 - 94)	65.1 (49 - 79)	0.73 (0.6 - 0.8)	2.3 (1.5 - 3.7)	0.3 (0.1 - 0.7)	59.5 (42 - 75)	84.9 (68 - 95)
Distinct fluorescence	59.3 (39 - 78)	95.4 (84 - 99)	0.77 (0.7 - 0.9)	12.7 (3.2 - 51.1)	0.4 (0.3 - 0.7)	88.9 (65 - 99)	78.9 (65 - 89)

## Discussion

Screening for cervical precancer has been shown to decrease incidence as well as mortality caused by cervical cancer (Sankarnarayanan et al., 2007). Several tests for screening are available with varying sensitivities and specificities, while no perfect test is introduced (Safaeian and Solomon, 2007). An ideal test not only should be highly sensitive in order not to miss any cancer or precancer but also highly specific in order to limit over-evaluation and over-treatment. The process of screening creates anxiety for the patients. In addition, inaccurate screening leads to over medicalization and increases healthcare costs which an already weak public health system cannot handle. 

In order to decrease over-treatment of screen positives, some triage or confirmatory test is usually done (Qiao et al., 2014). This strategy of testing in serial would decrease sensitivity and increase specificity. Testing in parallel and accepting either test being positive, such as in co-testing with cytology and HPV DNA, would increase sensitivity and decrease specificity. 

Testing in series is acceptable when treatments are fraught with serious complications. Confirmation of the diagnosis by colposcopy and biopsy would mean a two or three stage diagnostic work up wherein there could be substantial loss to follow up. Screening to prevent cancer is only as good as the treatment of screen positives. Thus, in low resource settings, a “screen and treat” strategy in a one-stop screening clinic is adopted to increase compliance to treatment (Jeronimo et al., 2016). In addition, “see and treat” or “screen and treat” strategies are in place to reduce total costs, with acceptance of over-treatment. If the treatment is innocuous, such as cryotherapy or thermocoagulation, this might be alright. However, loop electrosurgical procedures (LEEP) and conization can lead to complications like bleeding, infection, cervical stenosis, and preterm labor.

However, when resources are available and there is good follow up of women after a screening test, it is best to confirm the diagnosis before treatment. Rather than blind biopsies, directed biopsies of the cervix would yield accurate identification of existing cervical neoplasia that could otherwise be missed. Despite the availability of colposcopy, there is poor reproducibility, and colposcopically directed biopsies can miss 30 to 50% of cervical precancer (Stoler et al., 2011). Thus, colposcopy is enhanced with the use of acetic acid, Lugol’s iodine, endocervical curettage, scoring systems, and multiple random biopsies.

We conducted this study to explore the efficacy of FNa in targeting biopsies during colposcopy. Although biopsy is the gold standard, the accuracy of biopsy again depends on how well a cervical lesion is targeted. There was moderate agreement between distinct fluorescence and a Swede score of > 5 and FNa had a very high specificity. Thus, FNa could be used as an adjunct to colposcopy especially in young women desiring fertility or when Swede score is not very high.

It is important that mucus is removed before applying FNa as it gives a false fluorescence that has to be discounted. We found that FNa was moderately sensitive but highly specific and good at ruling in the presence of the disease when positive. Hence, if FNa is used in a diagnostic setting, it can better target sites for biopsy. Its high specificity can also be utilized in a screening setting, where over-referral for evaluation or over-treatment is a problem that needs to be overcome. The low sensitivity could be overcome by using Lugol’s iodine in parallel. However, like other visual methods, it is not able to detect endocervical lesions.

In the only published study on FNa in colposcopy by Moein et al., (2016), FNa was tested on 34 patients with no adverse reactions. In agreement with the current study, the aforementioned study showed that fluorescein sodium was more specific than acetic acid for identifying high-grade lesions in women with abnormal Pap smears. In low resource settings, FNa could be used in lieu of colposcopy to identify lesions for biopsy or LEEP. Even in colposcopy clinics, FNa could enhance the accuracy of colposcopically directed biopsies.

This study had a few limitations. Although we had 35 patients with CIN1+ lesions, only 27 had CIN2+ on biopsy. This resulted in wider confidence limits for diagnostic indices for CIN2+. Secondly, the digital documentation of fluorescence could have been independently graded by an investigator who was blind to the cytology and HPV results. 

The strengths of this work were that it was a prospective, well planned , had no verification bias. All colposcopic assessments and biopsies, after application of the various stains, were done by one investigator. FNa was compared to other screening methods. Although only distinct staining by VIA and VILI are considered positive in clinical use, we decided to compare two levels of staining as this was a research setting to assess the significance of faint staining by FNa.

It seems that FNa could be used in low resource settings along with VIA or VILI given that there was good agreement between distinct fluorescence and dense acetowhite staining. FNa increased the accuracy of VIA so it could be also used if colposcopy was not available. The use of FNa for targeting lesions in population-based screening programs needs to be assessed by future studies. The use of FNa has enough potential to deserve further study for early detection of cervical cancer.

In conclusion, the sensitivity of FNa was lower than iodine and Swede score >5, but specificity was as good as a Swede score >7. Given that it is low cost and easy to perform, FNa could be used to target cervical biopsies or, in a screen and treat program in low resource settings.

*Abbreviations and acronyms*

FNa: Fluorescent sodium

CIN: Cervical intraepithelial neoplasia

VIA: Visual inspection with acetic acid

VIAM: Visual inspection with acetic acid under magnification

VILI: Visual inspection with Lugol’s iodine

HPV: Human papillomavirus

DNA: Deoxyribonucleic acid

ROC: Receiver operating characteristic

SD: Standard deviation

PPV: Positive predictive value

NPV: Negative predictive value

BMI: Body mass index

LR: Likelihood ratio

HSIL: High grade squamous intraepithelial lesion

LEEP: Loop electrosurgical excision procedure

## Author Contribution Statement

The authors confirm contributions to the paper as follows: Study conception and design: SM, AP; Data collection: PB; Analysis and interpretation of results: TM, AP; Draft manuscript preparation: PB, AT, AP, SM; All authors reviewed the results and approved the final version of the manuscript.

## References

[B1] Bowring J, Strander B, Young M (2010). The Swede score: evaluation of a scoring system designed to improve the predictive value of colposcopy. J Low Genit Tract Dis.

[B2] Bray F, Ferlay J, Soerjomataram I (2018). Global cancer statistics 2018: GLOBOCAN estimates of incidence and mortality worldwide for 36 cancers in 185 countries. CA Cancer J Clin.

[B3] Chen B, Wang H, Ge P (2012). Gross total resection of Glioma with the intraoperative fluorescence-guidance of fluorescein sodium. Int J Med Sci.

[B4] Cipolla AF, Khedroo LG, Casella PA (1953). Fluorescein test for intraperitoneal rupture of the urinary bladder; experimental study. Surgery.

[B5] Haining WM (1966). Diagnostic value of intravenous fluorescein studies. Br J Ophthal.

[B6] Haynes WF Jr, Pittman FE, Christakis G (1960). Location of site of upper gastrointestinal tract hemorrhage by the fluorescein string test. Surgery.

[B7] Jeronimo J, Castle PE, Temin S (2016). Secondary prevention of cervical cancer: ASCO Resource-Stratified Clinical Practice Guideline. J Glob Oncol.

[B8] Kirchner FR, Proud GO (1960). Method for the identification and localization of cerebrospinal fluid, rhinorrhea and otorrhea. Laryngoscope.

[B9] Moein S, Mohajer R (2016). Fluorescein sodium as a contrast agent for colposcopy. Int J Gynaecol Obstet.

[B10] Qiao YL, Jeronimo J, Zhao FH (2014). Lower cost strategies for triage of human papillomavirus DNA-positive women. Int J Cancer.

[B11] Safaeian M, Solomon D (2007). Cervical cancer prevention – cervical screening: Science in evolution. Obstet Clin North Am.

[B12] Sankaranarayanan R, Basu P, Wesley RS (2004). Accuracy of visual screening for cervical neoplasia: Results from an IARC multicentre study in India and Africa. Int J Cancer.

[B13] Sankaranarayanan R, Esmy PO, Rajkumar R (2007). Effect of visual screening on cervical cancer mortality and incidence in Tamil Nadu, India: a cluster-randomised trial. Lancet.

[B14] Stoler MH, Vichnin MD, Ferenczy A (2011). FUTURE I, II and III Investigator The accuracy of colposcopic biopsy: analyses from the placebo arm of the Gardasil clinical trials. Int J Cancer.

[B15] Su X, Cheng K, Wang C (2013). Image-guided resection of malignant gliomas using fluorescent nanoparticles. Wiley Interdiscip Rev Nanomed Nanobiotechnol.

[B16] Van den Berg NS, van Leeuwen FW, van der Poel HG (2012). Fluorescence guidance in urologic surgery. Curr Opin Urol.

[B17] Xiao Z, Fangtian D, Rongping D (2010). Surgical management of epiretinal membrane in combined hamartomas of the retina and retinal pigment epithelium. Retina.

[B18] Yeh S, Wilson DJ (2010). Pars plana vitrectomy and endoresection of a retinal vasoproliferative tumor. Arch Opthalmol.

